# Development of a Flow Cytometric Immunoassay Using Recombinant Vascular Endothelial Growth Factor (VEGF)-Functionalized Poly(ε-Caprolactone) Microbeads for Bevacizumab Quantification

**DOI:** 10.34172/apb.46777

**Published:** 2026-07-11

**Authors:** Negar Namaei-Ghasemnia, Mohammad Rabiee, Fatemeh Davami, Yeganeh Talebkhan-Garoosi, Mohamad Ghorbani

**Affiliations:** ^1^Biomaterial Group, Department of Biomedical Engineering, Amirkabir University of Technology, Tehran, Iran; ^2^Biotechnology Research Center, Pasteur Institute of Iran, Tehran, Iran; ^3^Department of Molecular and Cellular Sciences, Faculty of Advanced Science and Technology, Tehran Medical Sciences, Islamic Azad University, Tehran, Iran

**Keywords:** Bevacizumab, Flow cytometry, Microbead-based flow cytometric immunoassays, Microbeads, Vascular endothelial growth factor

## Abstract

**Purpose:**

With the growing research interest in therapeutic monoclonal antibodies (mAbs) and their broad range of applications, the development of reliable analytical approaches for their quantification has become increasingly important. Since, microbead (MB)-based flow cytometric immunoassays have emerged as highly sensitive analytical methods, the aim of this study was to develop and evaluate a flow cytometry-based method using poly(ε-caprolactone) (PCL) MBs to quantify bevacizumab (BVZ) concentration.

**Methods:**

PCL MBs were synthesized through the emulsion/solvent evaporation method and modified by reaction with 1,2-ethylenediamine (EDA) to enable covalent binding of biomolecules. Based on the optimal biomolecule immobilization conditions determined using a model protein, purified recombinant vascular endothelial growth factor (VEGF) protein was immobilized on the surface of amine-functionalized MBs (A-MBs(45)) to capture BVZ, which was then detected using fluorescein isothiocyanate (FITC)-conjugated anti-human IgG antibody in flow cytometry.

**Results:**

The synthesized PCL MBs, with an average diameter of 6.62±0.17 µm, were within the suitable particle size range for immunoassay applications. The optimal aminolysis time was found to be 45 min at 37 °C, and the maximum protein coupling was achieved 5 h post-incubation at pH 8.0. The developed MB-based flow cytometric immunoassay demonstrated the potential to quantify BVZ with a limit of detection (LOD) and limit of quantification (LOQ) of about 6.71 and 22.73 ng mL-1, respectively.

**Conclusion:**

Considering the ease of fabrication and functionalization of PCL MBs under the obtained optimal conditions, along with their capability for analyte detection, these cost-effective analytical platforms may serve as promising candidates for BVZ quantification.

## Introduction

 Due to the rapid advancement of biological science, there is an urgent need for high-throughput analytical technologies to detect and quantify biomolecules in the field of drug discovery, medical diagnostics, and therapy.^1^ One remarkable example is the development of microbead (MB)-based flow cytometric assays, which provide significant advantages, including high sensitivity, specificity, flexibility, and precision, along with the capacity for simultaneous multiple-target detection in a single sample. Applications of MBs in flow cytometry predominantly rely on immunoassay formats for protein or antibody detection.^2-4^ Therefore, the surface of MBs needs to be functionalized with suitable biomolecules to capture the desired analytes.^5,6^

 MB-based flow cytometric immunoassays are widely regarded as an evolution of planar techniques such as enzyme-linked immunosorbent assay (ELISA), due to the reduced analysis time, lower sample consumption, reasonable cost, and faster binding kinetics resulting from the large surface area of MBs and the high concentration of detecting biomolecules on their surface, besides the ability for multiplexed detection.^7-9^

 Over recent decades, therapeutic monoclonal antibodies (mAbs) have emerged as the predominant treatment for various malignant, autoimmune, and inflammatory diseases.^10^ Despite the clinical benefits of mAb therapy, initial response rates exhibit considerable variability, ranging from 50 to 90%, and the response is often lost in a majority of patients over time, leading to disease progression, mainly as a consequence of unpredictable interindividual variability in mAb pharmacokinetics.^11-13^

 Bevacizumab (BVZ) is a recombinant humanized mAb administered to treat a broad range of solid malignancies such as metastatic colorectal cancer, non-small-cell lung cancer, ovarian cancer, metastatic breast cancer, glioblastoma multiforme, and renal cell carcinoma and also neovascular eye disorders.^14-17^ It binds to the secreted vascular endothelial growth factor (VEGF) and consequently inhibits VEGF-induced cell proliferation, leading to its anti-tumor activity via an anti-angiogenesis mechanism.^18-20^

 Several analytical methods, such as indirect ELISA^21^ and liquid chromatography-tandem mass spectrometry (LC-MS/MS),^22^ have been reported for BVZ quantification. However, LC-MS/MS methods are limited by time-consuming trypsin digestion and peptide purification steps, as well as complex pretreatment and analytical procedures.^23,24^ To address these limitations, Todoroki et al.^23^ proposed a methodology based on a combination of immunoaffinity magnetic purification and high-temperature reversed-phase LC (HT-RPLC), although its sensitivity was insufficient for low-concentration analysis. Moreover, alternative approaches, including a proximity extension assay based on anti-BVZ antibodies linked to specific oligonucleotide sequences,^25^ a mimotope-functionalized microporous alumina affinity membrane,^26^ and an aptamer-based fluorescence resonance energy transfer (FRET) assay,^27^ have been proposed for BVZ detection. Furthermore, Li et al.^28^ developed a LC-MS/MS-based strategy, in which an alternative sample pretreatment procedure was applied.

 Despite these advances, considerable research efforts are still directed toward the development of simple, sensitive, and reliable analytical platforms for BVZ quantification. In this context, the present study aimed to develop and optimize a MB-based flow cytometric immunoassay using VEGF-coated poly(ε-caprolactone) (PCL) MBs and examine its capability to quantify BVZ concentration as a first step toward future applications. To the best of our knowledge, the development of functionalized PCL MBs with suitable features as an analytical platform for BVZ quantification via flow cytometry has not been reported previously.

## Materials and Methods

###  Materials

 PCL pellets (average Mn 45 kDa), polyvinyl alcohol (PVA), 1,2-ethylenediamine (EDA), 2,2-dihydroxyindane-1,3-dione (ninhydrin, > 95%), 2-(N-morpholino) ethanesulfonic acid (MES), 1-ethyl-3-(3-dimethylaminopropyl) carbodiimide hydrochloride (EDC), N-hydroxysuccinimide (NHS), and bovine serum albumin (BSA) were purchased from Sigma-Aldrich, USA. Isopropyl-β-D-1-thiogalactopyranoside (IPTG) was sourced from Bio Basic, Canada. Tris(hydroxymethyl)aminomethane (Tris), bromophenol blue, sodium dodecyl sulfate (SDS), 2,2′,2′′,2′′′-(ethane-1,2-diyldinitrilo)tetraacetic acid (EDTA), N, N’-methylenebisacrylamide, Tween 20, 1,4-dioxane, sodium dihydrogen phosphate (NaH_2_PO_4_), sodium chloride (NaCl), and sulfuric acid (H_2_SO_4_) were obtained from Merck company. 3,3’,5,5’-Tetramethylbenzidine (TMB) substrate was purchased from Pishtazteb, Iran. Horseradish peroxidase (HRP)-conjugated anti-human IgG antibody produced in goat was supplied from AbD Serotec. FITC-conjugated anti-human IgG antibody (polyclonal rabbit anti-human IgG/FITC) was obtained from Dako, Denmark. BVZ (Avastin®, Roche) was provided by Farabi Hospital of Tehran, Iran. Rituximab (Rituxiver®) was purchased from Actoverco, Iran. A nickel-nitrilotriacetic acid (Ni-NTA) affinity column was procured from GE Healthcare, USA.

###  Preparation of PCL MBs 

 PCL was selected for the fabrication of MBs owing to its processing versatility and ease of surface modification.^29^ PCL MBs were synthesized via the emulsion/solvent evaporation method, which is the most commonly used technique to produce MBs with specific features from pre-existing polymer chains.^30-32^ Briefly, PCL was dissolved in dichloromethane to prepare a 2% w/v polymeric solution, which served as the oil phase. This solution was then emulsified in an aqueous phase containing 1% w/v PVA at a volume ratio of 1:5 for 10 min at 10000 rpm using a mechanical homogenizer (SR30; M-TOPS Co., Ltd., Yangju, Korea). The resultant emulsion was stirred gently for 4 h at room temperature to complete organic solvent evaporation. Finally, PCL MBs were collected by centrifugation at 1000 rpm for 2 min and washed with a copious amount of deionized water to remove surfactant. The MBs were observed under an optical microscope (OPTIKA MICROSCOPES, Italy).

###  Surface Modification of the MBs

 To enable subsequent immobilization of biomolecules, the MBs were aminolyzed in a 10% (v/v) solution of EDA/isopropyl alcohol at 37 °C in a water bath. Four different aminolysis times (15, 30, 45, and 60 min) were investigated. After aminolysis, the amine-functionalized samples (A-MBs(15), A-MBs(30), A-MBs(45), and A-MBs(60)) were rinsed with copious deionized water to remove residual EDA.

###  Ninhydrin Assay

 The ninhydrin assay was employed to determine the density of –NH_2_ groups on the surface of A-MBs(15), A-MBs(30), A-MBs(45), and A-MBs(60). After drying the samples at room temperature, 10 mg of each sample was dissolved in 1,4-dioxane and then treated with a 0.1 mol L^-1^ ninhydrin/ethanol solution for 20 min at 90 °C to accelerate the reaction between ninhydrin and –NH_2_ groups. The absorbance of the samples was measured at the wavelength corresponding to the highest absorbance (550 nm) using a UV–Vis spectrophotometer (Shimadzu UV–2550, Japan). The unmodified MBs sample was also evaluated as a control sample. To calculate the density of amine groups, a standard calibration curve was prepared using known concentrations of EDA in 1,4-dioxane with the addition of ninhydrin/ethanol solution.

###  Characterization of the MBs

####  Morphological Characterization

 The surface morphology of the synthesized MBs was observed under a scanning electron microscope (SEM; AIS2100, Secron Technology) at an accelerating voltage of 20 kV.

####  Particle Size Analysis (PSA)

 The MBs were analyzed using a particle size analyzer (Shimadzu SALD-2101, Japan) at a wavelength of 680 nm. The particles were dispersed in water and the particle size was assessed.

####  MBs Recovery

 The synthesized MBs were weighed after drying at 37 °C. By comparing the weight of MBs (W_MBs_) with the initial mass of PCL (W_PCL_), the recovery percentage was determined using the following equation:


(1) $$Recovery\;\%\left(MBs\right)=\frac{W_{MBs}}{W_{PCL}}\times 100$$

####  Fourier Transform Infrared (FTIR) Spectrum Analysis

 Unmodified MBs and A-MBs(45) were dried under ambient conditions and ground with KBr to form a homogeneous pellet. The infrared spectra of the samples were recorded using an FTIR spectrometer (NEXUS 670, Nicolet) in the range of 400–4000 cm^-1^. Spectral processing was performed using OMNIC software (version 6.0), and the spectra were combined using Origin software (version 95E).

###  Immobilization of a Model Protein on the Surface of A-MBs(45)

####  Calculation of A-MBs(45) Surface Saturation for a Model Protein

 After selecting 45 min as the optimal aminolysis time, protein-coupling experiments were conducted to investigate the potential of covalent immobilization of biomolecules on the surface of A-MBs(45). HRP-conjugated anti-human IgG antibody was used as a model protein because the presence of HRP allows easy and direct assessment of binding using a TMB substrate. The amount of protein required to saturate the surface of A-MBs(45) can be estimated using its molecular weight and relative affinity. Based on a protocol from Bangs Laboratories (TechNote 205: Covalent Coupling, Bangs Laboratories, Inc., Fishers, IN, USA), the following equation is recommended for calculating the amount of protein required to form a monolayer on the surface of the MBs.


(2) s=6ρDC

In which S is the amount of protein required to achieve surface saturation (mg of protein per gram of MBs), ρ and D represent the density (g cm^-3^) and mean diameter (μm) of the MBs, respectively, and C is the capacity of the MBs surface for a given protein (mg protein/m^2^ of sphere surface). Capacity data (C) for bovine IgG (150 kDa) and BSA (65 kDa) were determined by Cantarero et al.^33^ For other proteins, the surface saturation can be estimated by comparing their molecular weight to those of BSA and IgG. The calculated value from Equation 2 provides a starting point for identifying the optimal protein concentration. In some cases, such as low coupling efficiency, higher protein concentration may be required.

 Based on Equation 2, considering the density of PCL (1.145 g cm^-3^), the C constant for IgG (2.5 mg m^-2^), and the MBs average size of 6.62 µm, 1.98 mg of IgG per gram of MBs is required to achieve surface saturation. For a model protein concentration of 25 µg mL^-1^, the corresponding volume needed for a fixed amount of A-MBs(45) (0.5 mg) was calculated to be 40 µL. To investigate and ensure the surface saturation of the A-MBs(45), lower and higher mass ratios by applying lower and higher concentrations of the model protein in the constant calculated volume were also evaluated.

####  Investigating the Optimal Time and pH 

 The effects of model protein (HRP-conjugated anti-human IgG antibody) concentration, incubation time, and buffer pH on the amount of immobilized protein on the surface of the A-MBs(45) were examined. For each experimental condition, 0.5 mg of A-MBs(45) was suspended in 200 µL of 0.05 M MES buffer adjusted to pH of 5.0, 7.0, or 8.0. The model protein was diluted in the same MES buffer at corresponding pH values to prepare different concentrations of 12.5, 25, 50, and 75 µg mL^-1^. To activate the carboxyl groups of the model protein, 40 µL of each model protein concentration was incubated with 10 µL of freshly prepared crosslinking solution at 37 °C for 15 min (considering 1:100:100 protein:EDC:NHS molar ratio and EDC:NHS 1:1 volume ratio). After adding the EDC/NHS-activated model protein to A-MBs(45) suspensions prepared at the same pH, different incubation times (1, 5, and 16 h) were evaluated. Then all of the samples were rinsed twice with phosphate-buffered saline (PBS) containing 0.1% Tween 20 and once with PBS alone. After the addition of TMB and stopping the reaction with 2 M H_2_SO_4_, each sample was centrifuged, and 200 µL of its supernatant was transferred to a 96-well plate. The absorbance reading was performed by a microplate spectrophotometer (Epoch Biotech, Germany) at 450 nm. All the experiments were performed in triplicate. The optical absorbance of the supernatant from A-MBs(45), without incubation with the model protein, was also measured as a control sample after adding TMB and H_2_SO_4_.

####  Investigating the Importance of the Surface Modification

 After selecting the optimal pH (8.0) and incubation time (5 h), the effect of surface modification of MBs on biomolecule immobilization was evaluated by examining the binding of the EDC/NHS-activated model protein at different concentrations (6.25, 12.5, 25, 50, and 75 µg mL^-1^) onto A-MBs(45) and unmodified MBs, as described in previous section. All experiments were performed in triplicate.

###  VEGF Expression and Purification

 A colony of *Escherichia coli* BL21 (DE3) strain was transformed with a recombinant VEGF plasmid and cultured in 5 mL of Luria–Bertani (LB) broth medium. Protein expression was induced at the logarithmic phase (OD_600nm _= 0.4–0.6) by adding 0.5 mM IPTG, followed by incubation at 37 °C for 16 h. After the confirmation of protein expression with SDS–polyacrylamide gel electrophoresis (SDS–PAGE), large-scale protein expression was performed in 300 mL of LB medium. The bacterial pellet was resuspended in lysis buffer (10 mM EDTA, 100 mM NaCl, 50 mM Tris, 0.5% Triton X–100, 1 mg mL^-1^ lysozyme) and shaken for 1 h at room temperature. The suspension was subjected to ultrasonic agitation for 10 min, and the remaining cell debris was precipitated by centrifugation at 10000 rpm for 30 min at 4 °C. The processes of resuspension in lysis buffer, sonication, and centrifugation were repeated three times. The resulting inclusion bodies were resuspended in binding buffer (20 mM imidazole, 500 mM NaCl, 20 mM NaH_2_PO_4_, 8 M urea) and loaded onto an Ni-NTA affinity column. After washing the column with the washing buffer (50 mM imidazole, 500 mM NaCl, 20 mM NaH_2_PO_4_), the recombinant protein was eluted using elution buffer (500 mM imidazole, 500 mM NaCl, 20 mM NaH_2_PO_4_). The recombinant protein was refolded by dialysis against PBS buffer. Finally, the purification process was confirmed on a 12% SDS-PAGE, and the purified VEGF protein was stored at –20 °C for subsequent use.

###  Determination of VEGF Protein Concentration for Immobilization on A-MBs(45)

 By comparing the molecular weight of VEGF protein (16 kDa) to that of IgG (150 kDa) and considering the calculated mass ratio of IgG to MBs from Equation 2, the amount of VEGF protein required to achieve surface saturation was estimated to be 2.12 mg per gram of MBs. Therefore, the VEGF protein concentration was calculated to achieve surface saturation of 0.5 mg of A-MBs(45), considering a fixed protein volume of 40 µL. Moreover, an approximately 1.5-fold molar excess was also applied and investigated. Accordingly, VEGF protein at two concentrations, 26.5 and 40 µg mL^-1^, was tested. For the preparation of each sample (VEGF26.5-coated MBs and VEGF40-coated MBs), VEGF protein at the respective concentration in MES buffer was activated with EDC and NHS. The EDC/NHS-activated VEGF protein was then incubated with A-MBs(45) under the optimal time and pH. Subsequently, the samples were blocked with 1% BSA at 37 °C for 2 h to prevent non-specific binding to the surface of the A-MBs(45). Then, the samples were incubated with 200 µL of BVZ at different concentrations (0, 31.25, 62.5, 125, 250, 500, 1000, 2000, and 4000 ng mL^-1^) in MES buffer for 2 h at 4 °C, and the captured BVZ was detected using HRP-conjugated anti-human IgG antibody (1:8000). After each step, the samples were centrifuged, and the supernatants containing unbound molecules were removed. The samples were then intensively washed with PBS containing 0.1% Tween 20. Following a final wash with PBS, the TMB substrate was added, and the optical absorbance of the supernatant of each sample was measured at 450 nm. As a control, the absorbance of non-VEGF-coated MBs at the same BVZ concentrations was also determined.

###  Determination of FITC-Conjugated Anti-Human IgG Antibody Concentration for Flow Cytometric Analysis

 After selecting VEGF40-coated MBs as the analytical platform and following BVZ incubation as described in previous section, the use of FITC-conjugated anti-human IgG antibody at two concentrations of 5 and 50 µg mL^-1^ was evaluated for BVZ detection by flow cytometry. Data were acquired using a BD FACSCalibur flow cytometer (BD Biosciences, San Jose, CA, USA) and analyzed with FlowJo software (v10.4). Table 1 lists the encoded samples and their specifications. Sample 1 was evaluated as the control sample. Samples 2 and 4 were used to assess the non-specific binding of FITC-conjugated anti-human IgG antibody to VEGF40-coated MBs at each concentration. The optimal concentration of FITC-conjugated anti-human IgG antibody for flow cytometric analysis was determined by comparing the mean fluorescence intensities (MFIs) of different samples.

**Table 1:** The encoded samples and their specifications for optimizing the concentration of FITC-conjugated anti-human IgG antibody.

**Sample**	**VEGF concentration (µg mL**^-1^**)**	**BVZ concentration (ng mL**^-1^**)**	**FITC-conjugated anti-human IgG antibody concentration (µg mL**^-1^**)**
1	40	0	0
2	40	0	5
3	40	4000	5
4	40	0	50
5	40	4000	50

VEGF, Vascular endothelial growth factor; BVZ, Bevacizumab

###  Detection of BVZ Using Flow Cytometry

 BVZ at different concentrations of 0, 25, 50, 75, 125, 250, 500, 1000, 2000, 4000, 8000, and 16000 ng mL^-1^ was incubated with VEGF40-coated MBs (encoded samples BVZ0, BVZ25, BVZ50, BVZ75, BVZ125, BVZ250, BVZ500, BVZ1000, BVZ2000, BVZ4000, BVZ8000, and BVZ16000, respectively), and the captured BVZ was detected using FITC-conjugated anti-human IgG antibody at the concentration of 5 µg mL^-1^, as determined in previous section. After performing flow cytometry, the standard curve of MFI was plotted in terms of the BVZ concentration. The limit of detection (LOD) and limit of quantification (LOQ) were calculated as the concentrations corresponding to the background signal plus three- and ten-fold the standard deviation, respectively.^34^

 To evaluate the specific binding of BVZ to the immobilized VEGF on the surface of the VEGF40-coated MBs, the interaction of BVZ at a concentration of 16000 ng mL^-1^ with non-VEGF-coated MBs was also evaluated (sample: VEGF0BVZ16000). Moreover, to assess the selectivity and specificity of the designed assay, VEGF40-coated MBs were incubated with a non-specific antibody rituximab (RTX), anti-CD20 antibody, at a concentration of 16000 ng mL^-1^ prior to the addition of FITC-conjugated anti-human IgG antibody (sample: RTX16000).

###  Statistical Analysis

 Statistical analysis was performed using one-way analysis of variance (ANOVA). All quantitative results are expressed as mean ± standard deviation (SD) and considered statistically significant for P values < 0.05.

## Results and Discussion

###  Characterization of PCL MBs

 The optical microscopic image of the synthesized PCL MBs revealed that they did not aggregate by surface interaction (Figure 1a). The MBs recovery percentage was 78.09 by the Equation 1. The morphology and particle size of PCL MBs were examined with SEM and PSA, respectively (Figure 1b and 1c). The MBs were substantially spherical and uniform in shape, with an average diameter of 6.62 ± 0.17 µm. The results of the PSA test revealed that 90% of the MBs had sizes below 9.67 µm.

**Figure 1:**
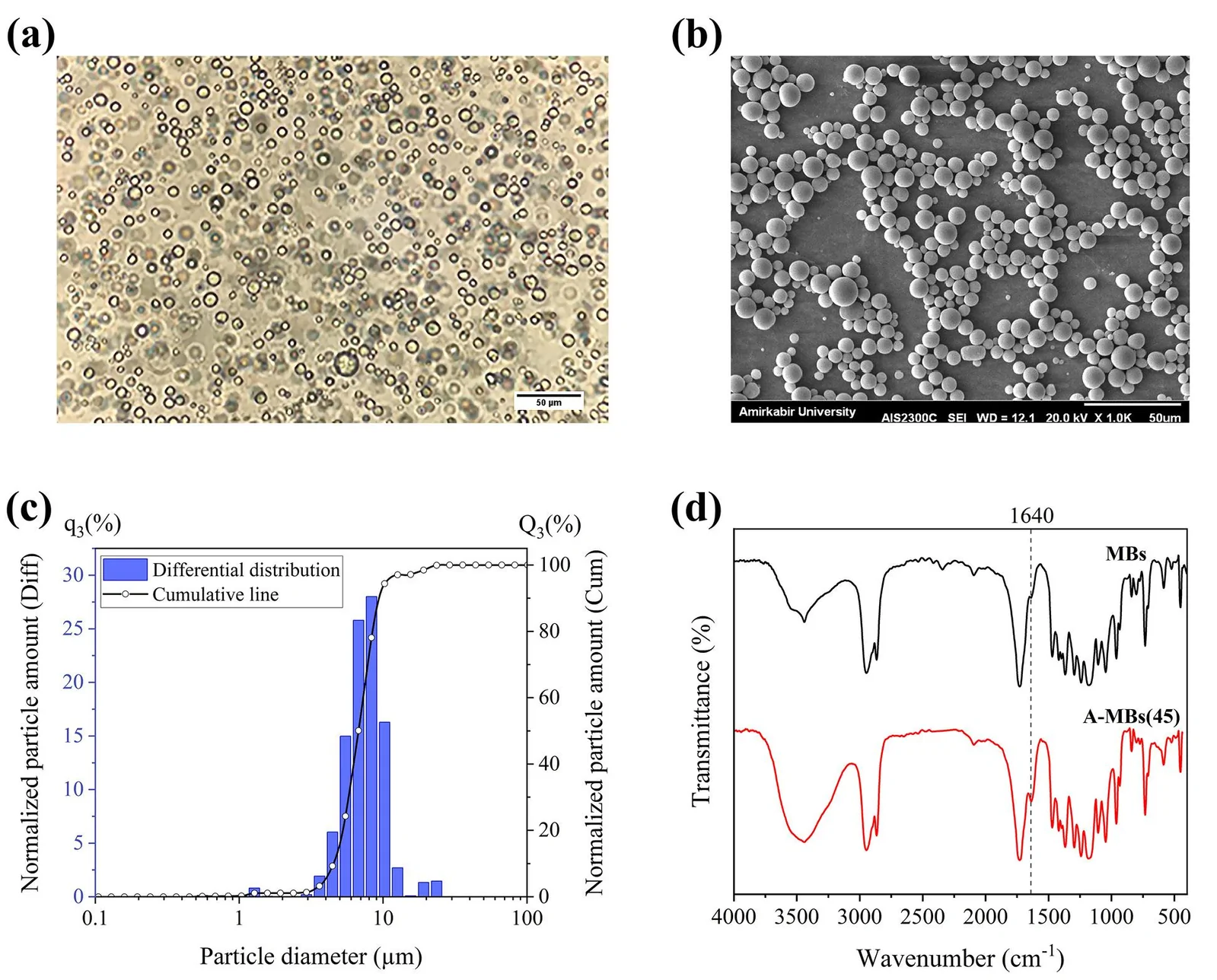
Characterization of PCL MBs. (a) Optical microscopic image, (b) SEM image, and (c) PSA of the synthesized MBs. (d) FTIR spectra of the unmodified MBs and A-MBs(45)

 For immunoassay applications, an optimal size range of MBs is typically between 0.5 and 9 µm. Smaller particles hinder reliable flow cytometric detection, and their limited surface area restricts biomolecule binding capacity, resulting in weak fluorescence signals. Conversely, larger particles require greater amounts of costly biochemical reagents for immobilization due to their extensive surface area. Moreover, at low analyte concentrations, fluorescence detection becomes more challenging as the fluorescent label is distributed over a larger surface. In addition, the increased sedimentation rate of larger particles during analysis may result in less uniform analyte binding.^35^ Accordingly, the synthesized PCL MBs had an appropriate size for use in MB-based flow cytometric immunoassays.

###  Surface Modification Results

 The surface modification of PCL MBs was performed via aminolysis with EDA. In this process, reaction between diamine and polyester backbone results in breaking ester bonds (–COO–), forming amide bonds (–CONH–), and introducing amine groups (–NH_2_) onto the surface, which makes the covalent attachment of biomolecules possible and provides a stable material-biomolecule conjugate.^36-39^ The following results demonstrated the successful occurrence of the aminolysis reaction.

####  FTIR

 FTIR spectra of the unmodified MBs and A-MBs(45) are presented in Figure 1d. The graphs were normalized to the PCL crystalline peak (1295 cm^-1^). Characteristic peaks of carbonyl and aliphatic groups at 1728 cm^-1^ (ν(C = O)), 2947 cm^-1^ and 2866 cm^-1^ (ν_as_(C–H) and ν_s_(C–H)), as well as peaks at 1295 cm^-1^ and 1180 cm^-1^ that are respectively related to the tensile state of CO and CC bonds in the crystalline and amorphous phases, were observed in both unmodified MBs and A-MBs(45) samples. Peaks that are related to amide bonds were expected to appear in the A-MBs(45) sample at 1640 cm^-1^ and 3320 cm^-1^ (ν(C = O) and ν(N–H)). The peak at 1640 cm^-1^ confirmed the formation of amide bonds. The peak spreading at the range of 3320 cm^-1^ could be attributed to the overlapping of N–H stretching with the bonds of water in the A-MBs(45) sample.^36,40-42^ The FTIR results revealed that amine functional groups were successfully introduced on the surface of the MBs in the aminolysis process (Figure 1d).

####  Ninhydrin Assay

 The ninhydrin assay was performed to investigate the effect of the aminolysis time on the formation of amine groups on the surface of MBs. The images of unmodified MBs and amine-functionalized MBs (A-MBs(15), A-MBs(30), A-MBs(45), and A-MBs(60)) after the ninhydrin assay are shown in Figure 2a. The developed purple-red color in amine-functionalized MBs is due to the reaction between ninhydrin and amine groups and indicates the presence of amine groups in these samples. The graph of the absorbance at 550 nm against the aminolysis time and the plotted calibration curve of the absorbance values at 550 nm as a function of NH_2_ concentration are presented in Figure 2b and 2c, respectively. The density of amine groups on the surface of A-MBs(15), A-MBs(30), A-MBs(45), and A-MBs(60) was determined. By increasing the aminolysis time, the density of amine groups on the surface of PCL MBs increased and reached a maximum value in 45 min (A-MBs(45)), equaling 5.980 × 10^-7^ mol mg^-1^. After 45 min of aminolysis, a decrease in the density of amine groups was observed (Figure 2b), which may be attributed to chain scission, the formation of oligomers, and the removal of other low-mass fragments throughout the reactionand rinsing steps.^40,43^ This result is in line with the previous findings. Yuan et al. and Balmayor et al. also employed aminolysis to immobilize gelatin or protein onto PCL substrates, and they observed that prolonging reaction time caused a decrease in the number of amine groups. They reported that the density of amine groups on the PCL surface reached a maximum value after 1 h of exposure to 1,6-hexanediamine.^40,44^ Since smaller diamine molecules such as EDA have been shown to accelerate the aminolysis reaction due to lower steric hindrance and better penetration ability,^45,46^ it was expected to achieve maximum density of amine groups in a shorter period of time (45 min).

**Figure 2:**
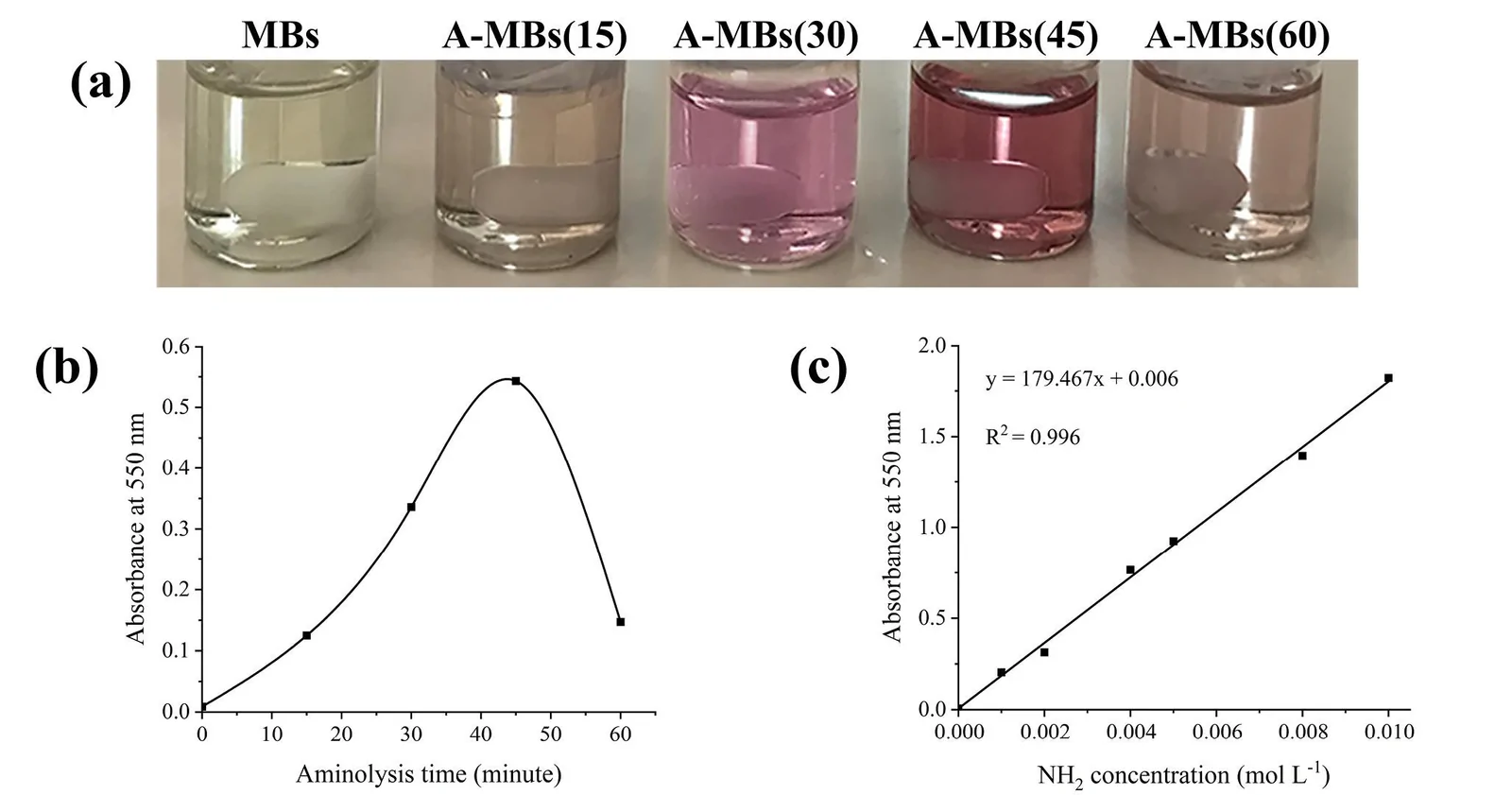
Ninhydrin assay. (a) Image of the unmodified MBs and amine-functionalized MBs with different aminolysis times of 15, 30, 45, and 60 min (A-MBs(15), A-MBs(30), A-MBs(45), and A-MBs(60)) treated with ninhydrin. (b) The absorbance at 550 nm of the samples as a function of aminolysis time in the ninhydrin assay. (c) Calibration curve of the reaction ninhydrin-NH2

###  Protein Immobilization Results

 Biomolecule immobilization on analytical platforms is the most critical step that directly impacts the analytical performance.^47^ Among various strategies, methods based on covalent binding have attracted considerable attention due to enabling highly stable bonds, preventing desorbing or leaching of biomolecules over time. EDC/NHS-mediated heterobifunctional crosslinking of biomolecules through their amine or carboxyl groups to free carboxyl or amine groups on bioanalytical platforms has been extensively used for assay development due to its ability to preserve the bioactivity of immobilized biomolecules.^48,49^ EDC initially activates the carboxyl functional groups, forming an unstable *O*-acylisourea intermediate that is prone to rapid hydrolysis. To increase the stability of the active intermediate and enhance the coupling efficiency, the reaction is often carried out in the presence of NHS. NHS converts the *O*-acylisourea intermediate into NHS ester, which can be readily displaced by primary amine groups through nucleophilic attack, resulting in the formation of covalent amide bonds.^50-53^ In this study, the attachment of EDC/NHS-activated model protein to amine groups introduced on the surface of A-MBs(45) was evaluated to determine the optimal immobilization conditions.

####  Optimal Time and pH for Protein Immobilization

 The optical absorbance, which reflects the amount of immobilized EDC/NHS-activated model protein on the surface of A-MBs(45) after incubation at various pH values (5.0, 7.0, and 8.0) and incubation times (1 h, 5 h, and 16 h), is shown in Figure 3a–c. It should be noted that when the model protein was not added to A-MBs(45), no optical absorbance was detected. As expected, incubation of A-MBs(45) with EDC/NHS-activated model protein at pH 5.0, compared to pH 7.0 and 8.0, resulted in lower absorbance at each incubation time, indicating a reduced amount of immobilized protein. This can be attributed to the increased number of protonated amine groups (NH_3_^+^) on the surface of A-MBs(45), which are not involved in the coupling reaction due to their poor nucleophilic capability.^44,54^ Moreover, at pH 5.0, the surface charge of the A-MBs(45) is positive (NH_3_^+^), and the protein is also positively charged. Therefore, electrostatic repulsion can be another reason for the reduced protein binding to the surface of the A-MBs(45).^44^

**Figure 3:**
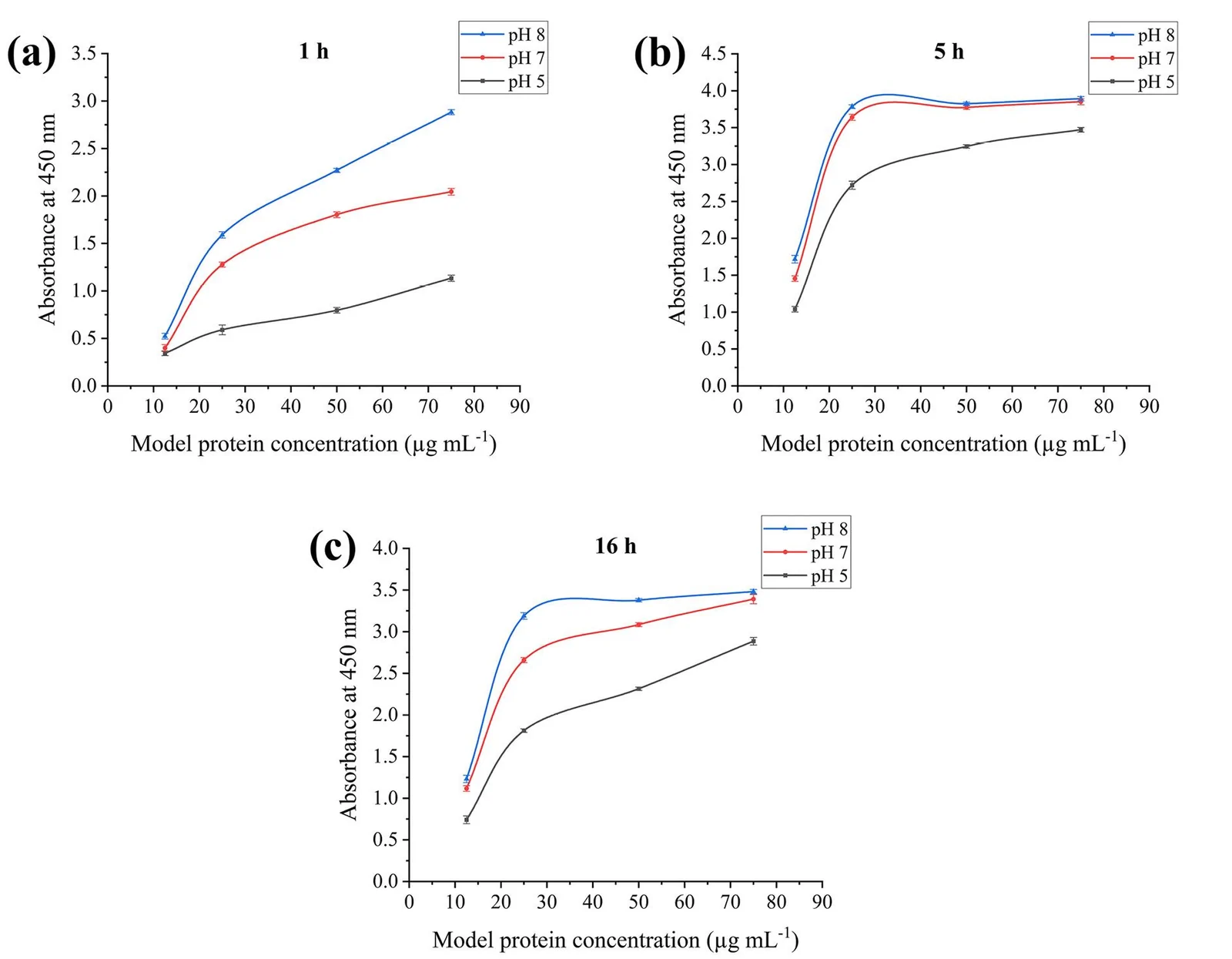
The 450 nm absorbance of the supernatants from A-MBs(45) incubated with different concentrations of EDC/NHS-activated model protein (HRP-conjugated anti-human IgG antibody). (a) 1 h, (b) 5 h, and (c) 16 h incubation at different coupling pH values (5.0, 7.0, and 8.0). The results are shown as the average value ± SD, n = 3

 At the end of each incubation time, the maximum protein binding was observed at pH 8.0, which was therefore considered the optimal pH for protein immobilization on the surface of A-MBs(45). This result is consistent with previous reports that the immobilization of biomolecules on amine-functionalized surfaces using EDC-based strategies is more efficient at weakly alkaline pH, typically in the range of 7.0–8.0, whereas further increasing the buffer pH accelerates the hydrolysis of the NHS ester, leading to less efficient crosslinking.^48,53,55^ As shown in Figure 3b, the absorbance values of A-MBs(45) incubated with different concentrations of EDC/NHS-activated model protein at pH 7.0 and 8.0 after 5 h are close to each other. Therefore, it can be assumed that with a further increase in the coupling pH at this incubation time, the rate of NHS ester hydrolysis will probably be higher than the rate of its reaction with the amine groups, which leads to lower biomolecule immobilization.

 From the incubation time analysis, it can be inferred that 1 h of incubation was not enough for complete binding of the model protein to the surface of A-MBs(45), while applying 16 h incubation resulted in a decrease in the optical absorbance of all samples. This may be due to surface degradation and the separation of the biomolecules from the surface. It can be concluded that, at the same pH and for each model protein concentration, 5 h incubation with A-MBs(45) resulted in the highest absorbance value.

Figure 3a, which corresponds to 1 h incubation time, shows that at different pH values the graphs continue to rise, indicating that the surface of A-MBs(45) has not been saturated even at the model protein concentration of 75 µg mL^-1^. However, after 5 h of incubation at pH 7.0 and 8.0, no increase in absorbance values was observed when the model protein concentration was increased from 25 µg mL^-1^ (Figure 3b). The constant trend of these graphs from the model protein concentration of about 25 µg mL^-1^, indicates that there were no more available amine groups for covalent coupling of additional molecules. So, the surface saturation of the A-MBs(45) was achieved at the calculated value from Equation 2, demonstrating the high coupling efficiency.

####  The Importance of Surface Modification in Protein Immobilization

 The absorbance at 450 nm for A-MBs(45) and unmodified MBs, which were exposed to different concentrations of EDC/NHS-activated model protein at pH 8.0 for 5 h, is shown in Figure 4. It is evident that the absorbance of the supernatants from A-MBs(45) incubated with each model protein concentration was higher than that of the unmodified MBs, indicating a higher amount of immobilized protein on the surface of A-MBs(45). According to the graph, the absorbance value from the supernatant of A-MBs(45) incubated with EDC/NHS-activated model protein at a concentration of 6.25 µg mL^-1^ was approximately equal to the absorbance value of the unmodified MBs incubated with a concentration of 75 µg mL^-1^ under the same conditions. It means that to achieve the same protein density created on the surface of A-MBs(45) with a model protein concentration of 6.25 µg mL^-1^, the unmodified MBs must be incubated with a 12-fold higher protein concentration. Therefore, the importance of surface modification for biomolecule immobilization can be understood. In the absence of functional groups on the surface of MBs, biomolecule binding is weak and non-covalent and can be lost during the washing process. In contrast, when the surface is modified with suitable functional groups for conjugation, covalent bonds can be established between the biomolecules and surface, ensuring the stability of the immobilized biomolecules.

**Figure 4:**
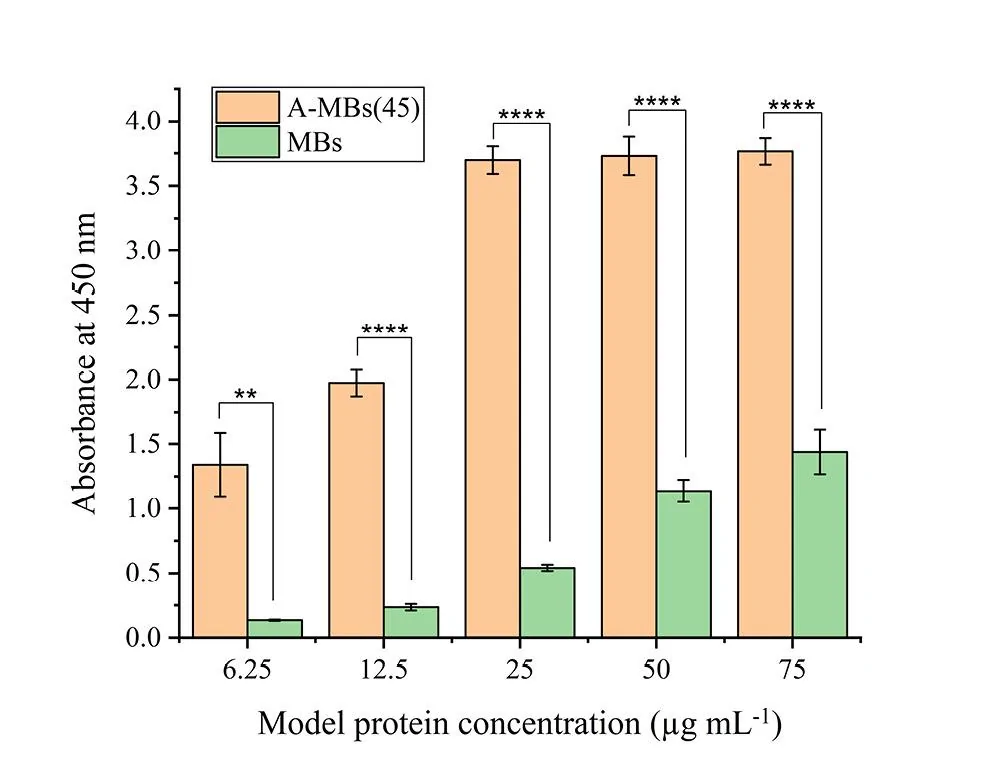
The 450 nm absorbance of the supernatants from unmodified MBs and A-MBs(45) incubated with different concentrations of EDC/NHS-activated model protein (HRP-conjugated anti-human IgG antibody) at pH 8.0 for 5 h. The results are shown as the average value ± SD, n = 3, **P < 0.01, ****P < 0.0001

###  SDS–PAGE Analysis of Purified Recombinant VEGF Protein

 The recombinant VEGF protein was detected by a Coomassie-stained SDS–PAGE as a single band at 16 kDa (Figure 5).

**Figure 5:**
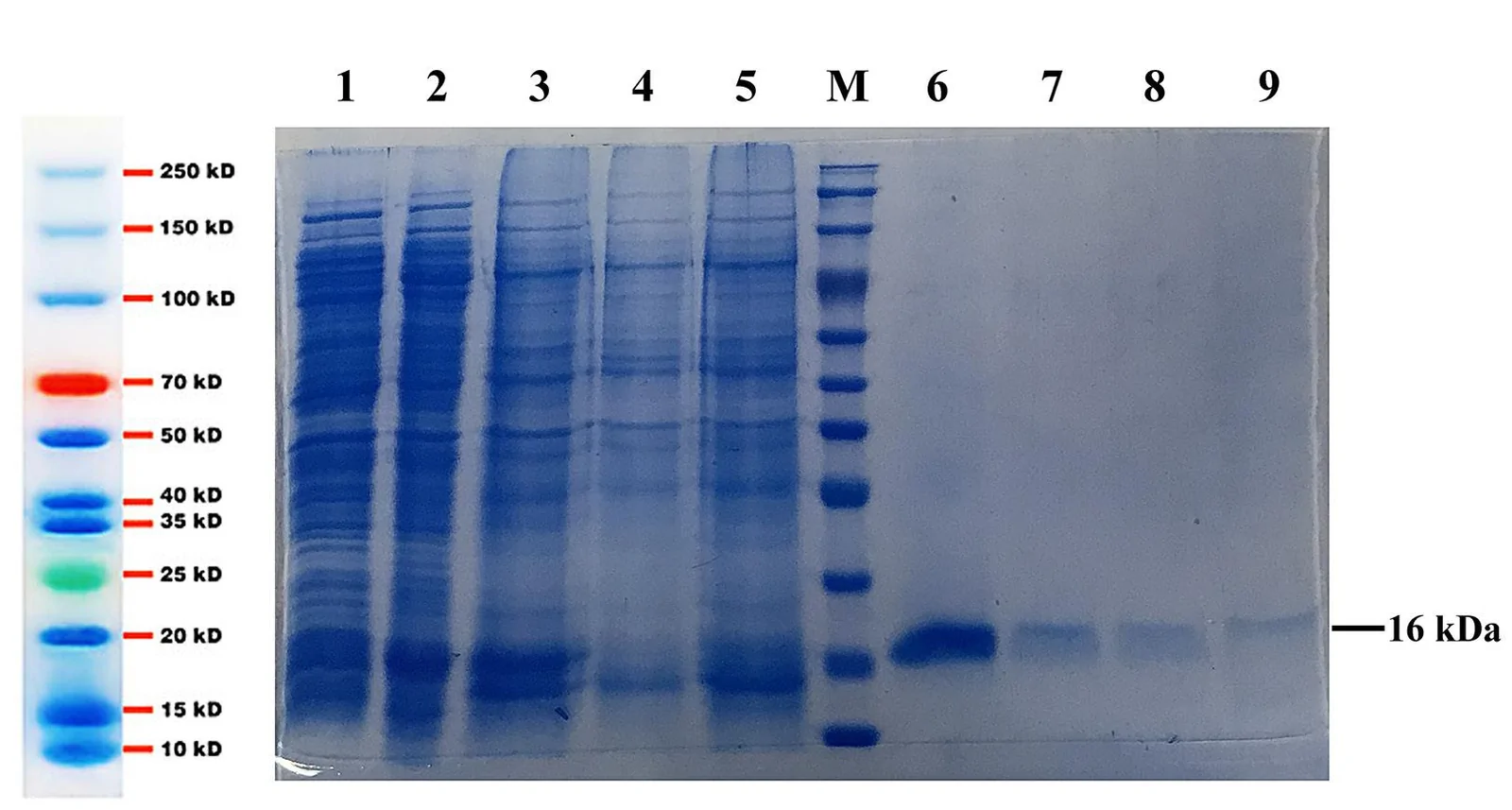
SDS-PAGE analysis of the purified VEGF protein. Lanes 1 and 2: Recombinant E. coli debris before and after induction; Lane 3: Initial sample; Lanes 4 and 5: Flow through samples; M: Protein MW marker; Lanes 6 to 9: Elution fractions containing the purified protein (0.074, 0.038, 0.028, and 0.012 mg mL-1, respectively)

###  Optimal Concentration of VEGF for Immobilization on the Surface of the A-MBs(45)

 The results of the absorbance measurements of the supernatants derived from the incubation of non-VEGF-coated MBs, VEGF26.5-coated MBs, and VEGF40-coated MBs with different concentrations of BVZ, after adding HRP-conjugated anti-human IgG antibody and TMB substrate, are shown in Figure 6. As illustrated, in the case of non-VEGF-coated MBs, the optical absorbance was about 0.2, and increasing the concentration of BVZ did not lead to any significant increase in the optical absorbance (*P* > 0.05). In the case of VEGF-coated MBs (at concentrations of 26.5 or 40 µg mL^-1^), the absorbance values increased following the increase in the BVZ concentration, confirming the successful immobilization of VEGF on the surface of A-MBs(45) and indicating specific binding of BVZ to the immobilized VEGF.

**Figure 6:**
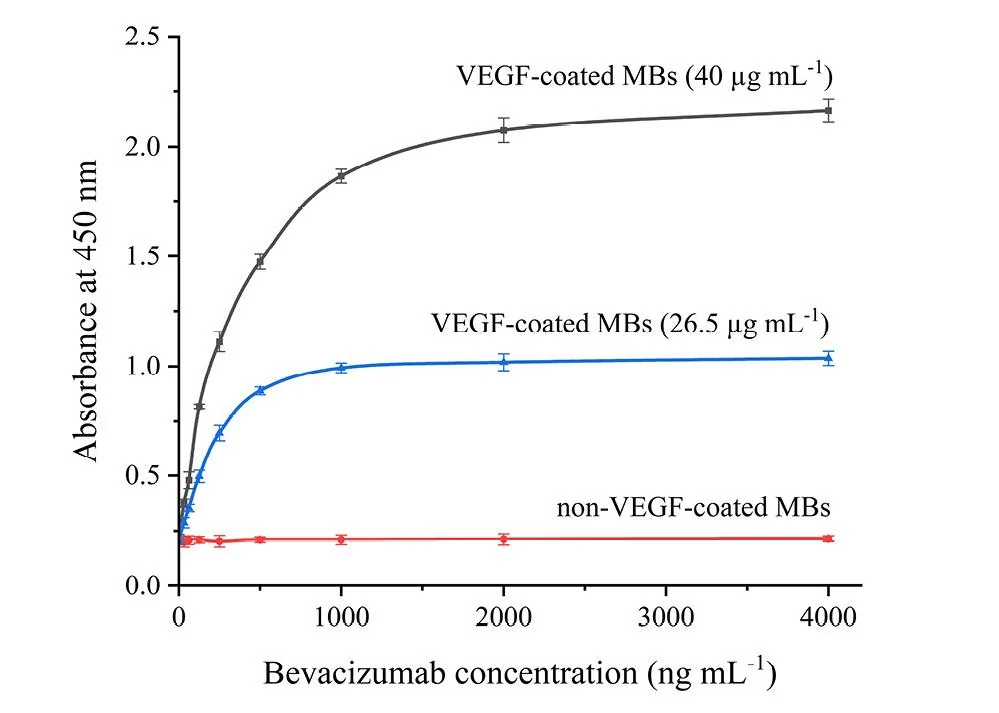
The 450 nm absorbance of supernatants from VEGF26.5-coated MBs, VEGF40-coated MBs, and non-VEGF-coated MBs incubated with different concentrations of BVZ and 1:8000-diluted HRP-conjugated anti-human IgG antibody. The results are shown as the average value ± SD, n = 3

 When VEGF at a concentration of 26.5 µg mL^-1^ was immobilized on the surface of A-MBs(45), by enhancing the BVZ concentration beyond 1000 ng mL^-1^, no significant increase in the absorbance (*P* > 0.05) was observed, indicating surface saturation. The higher slope of the linear region of the graph corresponding to VEGF40-coated MBs implies a higher sensitivity in comparison with VEGF26.5-coated MBs. Because a slight change in the concentration of BVZ in the linear region resulted in a great difference in the absorbance, which directly impacts the accuracy and sensitivity of the method. Therefore, a VEGF concentration of 40 µg mL^-1^ was selected for immobilization on the surface of A-MBs(45) (Figure 6).

###  Optimal Concentration of FITC-Conjugated Anti-Human IgG Antibody in Flow Cytometric Analysis

 The MFI values of samples 1 to 5 that are listed in Table 1 were 11.3, 69.3, 978, 94.8, and 1354, respectively. Sample 1 was tested as a negative control to confirm that VEGF40-coated MBs exhibited no intrinsic fluorescent properties. As expected, this sample had a very low MFI compared to the other samples. The MFIs of the control samples 2 and 4 were evaluated to investigate the non-specific binding of FITC-conjugated anti-human IgG antibody to the surface of VEGF40-coated MBs. In both series of samples treated with 5 and 50 µg mL^-1^ of FITC-conjugated anti-human IgG antibody concentrations, a displacement of the peaks to the right was observed in the samples incubated with BVZ at a concentration of 4000 ng mL^-1^ (samples 3 and 5), compared to those incubated in the absence of BVZ (samples 2 and 4), demonstrating a pronounced difference in the MFIs of these samples (Figure 7). This discrepancy confirms that the high MFIs of samples 3 and 5 resulted from the specific binding of FITC-conjugated anti-human IgG antibodies to the captured BVZ, rather than from their non-specific binding to the surface of VEGF40-coated MBs.

**Figure 7:**
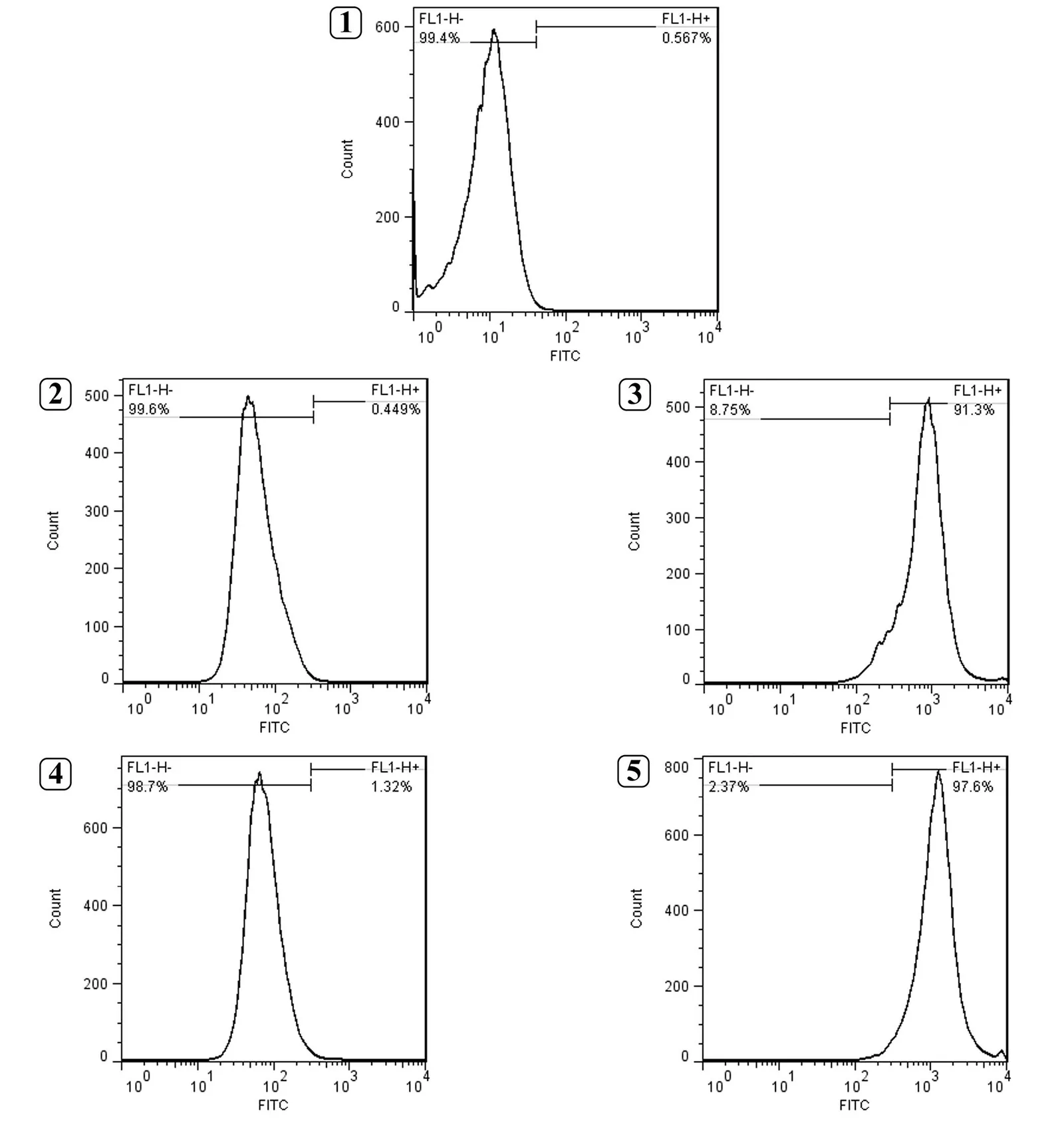
Flow cytometry histograms of samples 1 to 5 that are listed in Table 1. Sample 1: VEGF40-coated MBs as a control sample; Sample 2: VEGF40-coated MBs incubated with 5 µg mL-1 of FITC-conjugated anti-human IgG antibody; Sample 3: VEGF40-coated MBs incubated with 4000 ng mL-1 of BVZ and 5 µg mL-1 of FITC-conjugated anti-human IgG antibody; Sample 4: VEGF40-coated MBs incubated with 50 µg mL-1 of FITC-conjugated anti-human IgG antibody; Sample 5: VEGF40-coated MBs incubated with 4000 ng mL-1 of BVZ and 50 µg mL-1 of FITC-conjugated anti-human IgG antibody

 It was observed that increasing the FITC-conjugated anti-human IgG antibody concentration increased the MFI of both the control sample and the sample exposed to a certain concentration of BVZ in the same proportion. Therefore, although the MFI of sample 5 was higher than that of sample 3, the MFI ratios of samples 3 to 2 and 5 to 4 were similar. This indicates that similar results can be obtained using FITC-conjugated anti-human IgG antibody concentrations of either 5 or 50 µg mL^-1^. By considering the cost of the conjugated antibodies, a concentration of 5 µg mL^-1^ of FITC-conjugated anti-human IgG antibody was selected as the appropriate concentration.

###  BVZ Quantification Results

 The MFI values of the samples BVZ0 to BVZ16000 are listed in Table 2.

**Table 2:** The encoded samples and their corresponding MFI values following incubation of VEGF40-coated MBs with different concentrations of BVZ.

**Sample**	**BVZ concentration ** **(ng mL**^-1^**)**	**MFI ** **(mean±SD)**
BVZ0	0	6.16 ± 0.19
BVZ25	25	8.26 ± 0.28
BVZ50	50	10.23 ± 0.38
BVZ75	75	13.15 ± 1.05
BVZ125	125	16.71 ± 0.51
BVZ250	250	26.63 ± 0.88
BVZ500	500	41.50 ± 2.70
BVZ1000	1000	57.05 ± 1.05
BVZ2000	2000	72.05 ± 1.85
BVZ4000	4000	89.59 ± 2.81
BVZ8000	8000	106.50 ± 3.50
BVZ16000	16000	122.85 ± 1.15

BVZ, Bevacizumab; MFI, Mean fluorescence intensity

 Examination of samples BVZ0 to BVZ16000 showed increased MFIs of the samples by increasing the BVZ concentration. This can also be deduced from the displacement of the histograms by increasing the concentration of BVZ to higher fluorescence intensities (Figure 8a). Statistical analysis revealed that the MFIs of samples BVZ0–BVZ16000 were significantly different (*P* < 0.05), representing the ability of the assay to quantify BVZ concentrations within the range of 0–16000 ng mL^-1^. The standard curve of MFI as a function of BVZ concentration is shown in Figure 8b, which can be used to determine BVZ concentration in unknown samples. The continuous increasing trend without reaching a plateau demonstrated that the available active binding sites on the surface of VEGF40-coated MBs were not saturated even at a BVZ concentration of 16000 ng mL^-1^. The developed MB-based flow cytometric immunoassay exhibited a good sensitivity and linearity in the BVZ concentration range of 0–250 ng mL^-1^ (R^2^ = 0.999), and the calculated values for LOD and LOQ were almost 6.71 and 22.73 ng mL^-1^, respectively. It should be noted that sample dilution to this linear range leads to more precise BVZ quantification, since a small change in concentration produces a more pronounced change in MFI.

**Figure 8:**
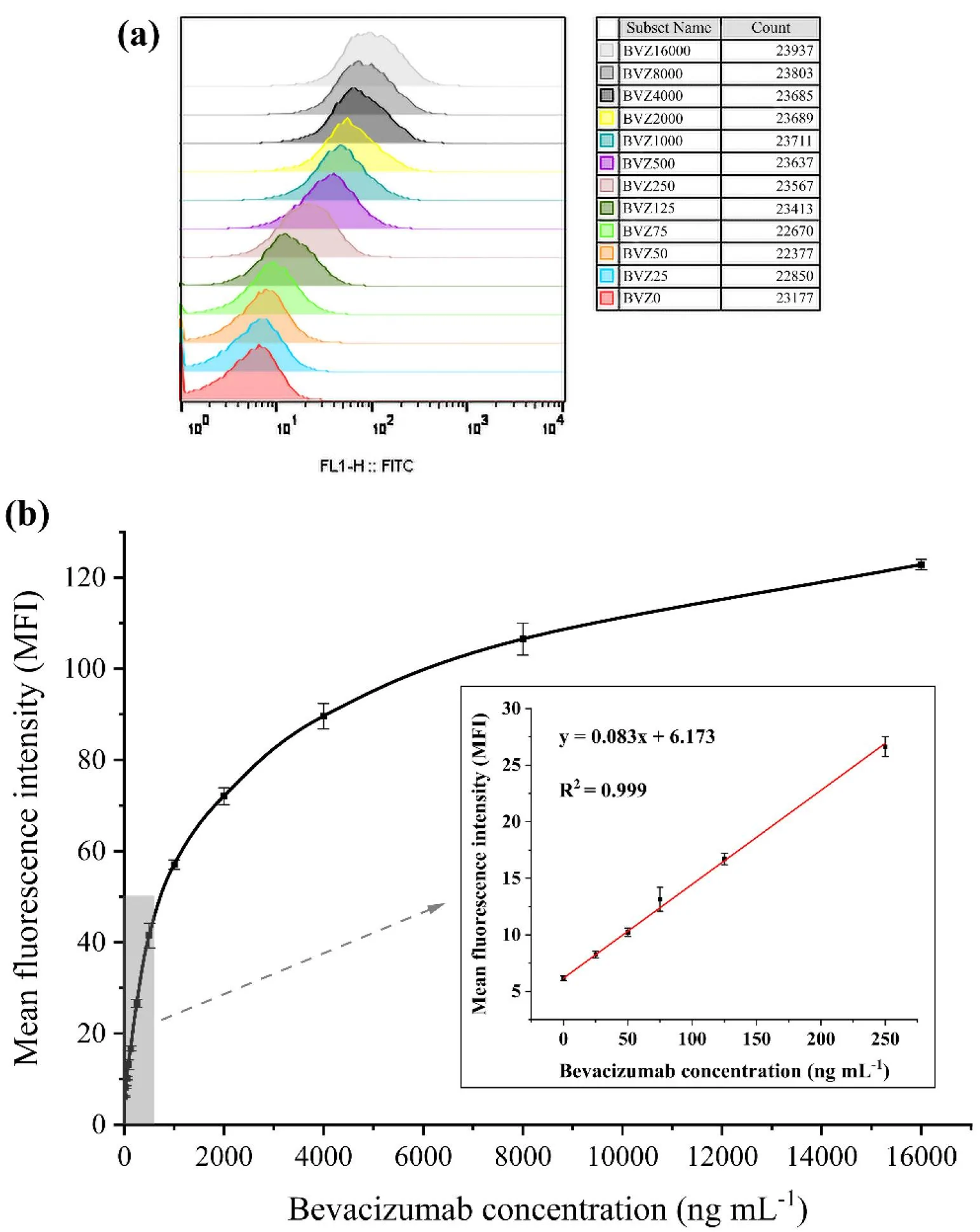
(a) Histograms of flow cytometry analysis of samples BVZ0 to BVZ16000; VEGF40-coated MBs incubated with BVZ at different concentrations of 0, 25, 50, 75, 125, 250, 500, 1000, 2000, 4000, 8000, and 16000 ng mL-1, respectively. The binding of BVZ to VEGF40-coated MBs was detected by FITC conjugated anti-human IgG antibody at a concentration of 5 µg mL-1. (b) The standard curve of the MFI as a function of BVZ concentration. The results are shown as the average value ± SD, n = 3

 At the same BVZ concentration of 16000 ng mL^-1^, the significantly higher MFI of sample BVZ16000 (122.85 ± 1.15) compared with sample VEGF0BVZ16000 (8.47 ± 0.042) affirmed the specific binding of BVZ to VEGF protein on the surface of VEGF40-coated MBs, because non-VEGF-coated MBs (sample VEGF0BVZ16000) showed low affinity to capture BVZ for subsequent detection.

 Comparing the MFIs of samples BVZ16000 and RTX16000 indicated that the MFI of sample BVZ16000 (122.85 ± 1.15) was approximately fifteen times higher than that of sample RTX16000 (8.11 ± 0.13). By this result, the selectivity of the assay and its specific function for the detection of BVZ can be concluded.

 This study demonstrated the potential of the developed MB-based flow cytometric immunoassay for BVZ quantification. By comparing the results obtained in this study with previous studies, it can be concluded that the designed assay, employing VEGF-coated PCL MBs with the LOQ of 22.73 ng mL^-1^, could be considered as a simple and cost-effective candidate for measuring BVZ concentration. Nevertheless, two main limitations should be acknowledged. First, the binding efficiency was not evaluated through measurement of the unbound BVZ remaining in the supernatant after incubation with VEGF40-coated MBs. Second, the assay was evaluated only in a simple aqueous matrix rather than in complex biological specimens. Therefore, further studies are essential to evaluate the ability of the proposed immunoassay to measure BVZ levels in biological specimens.

 It is anticipated that this analytical platform could be adapted to quantify various analytes by immobilizing their specific recognition elements on the surface of A-MBs(45) under the optimized biomolecule immobilization conditions obtained in this study.

## Conclusion

 In summary, this work reports a MB-based flow cytometric immunoassay for quantification of BVZ. We have developed and characterized PCL MBs as an inexpensive solid support for the immobilization of recombinant VEGF. This method, by combining the advantages of MBs and flow cytometry, exhibited acceptable sensitivity and specificity. As a next step, it is recommended that the performance of the proposed MB-based flow cytometric immunoassay be evaluated in complex biological samples. Moreover, measuring the unbound BVZ in the supernatant and performing mass balance calculations is suggested to fully validate the binding efficiency. Furthermore, regarding the capability of the functionalized PCL MBs and the obtained optimal conditions for biomolecule immobilization and analyte detection, the proposed methodology can be extended to develop assays for other protein-based therapeutics and biomarkers, underscoring its broad applicability in pharmaceutical analysis and clinical diagnostics.

## Competing Interests

 The authors declare that they have no conflicts of interest.

## Ethical Approval

 Not applicable.
